# The FSHD jigsaw: are we placing the tiles in the right position?

**DOI:** 10.1097/WCO.0000000000001176

**Published:** 2023-06-14

**Authors:** Valentina Salsi, Gaetano Nicola Alfio Vattemi, Rossella Ginevra Tupler

**Affiliations:** a Department of Biomedical, Metabolic and Neural Sciences, University of Modena and Reggio Emilia, Modena; b Department of Neurosciences, Biomedicine and Movement Sciences, Section of Clinical Neurology, University of Verona, Verona, Italy; c Department of Molecular Cell and Cancer Biology; d Li Weibo Institute for Rare Diseases Research at the University of Massachusetts Medical School, Worcester, USA

**Keywords:** biomarkers, clinical phenotype, DUX4, epigenetics, facioscapulohumeral muscular dystrophy, magnetic resonance imaging, therapeutic approaches

## Abstract

**Purpose of review:**

Facioscapulohumeral muscular dystrophy (FSHD) is one of the most common myopathies, involving over 870,000 people worldwide and over 20 FSHD national registries. Our purpose was to summarize the main objectives of the scientific community on this topic and the moving trajectories of research from the past to the present.

**Recent findings:**

To date, research is mainly oriented toward deciphering the molecular and pathogenetic basis of the disease by investigating DUX4-mediated muscle alterations. Accordingly, FSHD drug development has been escalating in the last years in an attempt to silence DUX4 or to block its downstream effectors. Breakthroughs in the field include the awareness that new biomarkers and outcome measures are required for tracking disease progression and patient stratification. The need to develop personalized therapeutic strategies is also crucial according to the phenotypic variability observed in FSHD subjects.

**Summary:**

We analysed 121 literature reports published between 2021 and 2023 to assess the most recent advances in FSHD clinical and molecular research.

## INTRODUCTION

Facioscapulohumeral muscular dystrophy (FSHD) (MIM 158900), one of the most common myopathies, has been considered a fully penetrant autosomal dominant disease associated with deletions of integral copies of the tandemly arrayed D4Z4 repeat at chromosome 4q35 [1]. FSHD is the only human disease causally linked to Copy Number Variation of macrosatellite deoxyribonucleic acid (DNA) elements [2]. At present, the pathogenesis of FSHD1, accounting for about 95% of cases, is explained by a model that involves the loss of epigenetic silencing and aberrant expression of *DUX4*, a retrogene embedded within the D4Z4 array [3]. In rarer FSHD2 cases, *DUX4* expression is associated with global CpG reduced methylation of the D4Z4 array that is usually caused by defects in genes encoding for proteins involved in epigenetic suppression, that is, the structural maintenance of chromosomes flexible hinge domain containing 1 gene (*SMCHD1*) [4], the methyltransferase 3B gene (*DNMT3B*) [5] and the ligand-dependent nuclear receptor interacting factor 1 gene (*LRIF1*) [6,7],

Although the etiology of the disease has been attributed to gain-of-toxic function stemming from the aberrant expression of DUX4, the exact pathogenic mechanisms involved in muscle wasting remain to be elucidated. This is because clinical and epidemiological data do not mirror the idea of a Mendelian disease in terms of penetrance and inter/intra familiar phenotypic variability [8–14]. Several intersections are observed between molecular data and various clinical phenotypes, including healthy people carrying the same molecular signature as affected individuals [15–17]. Therefore, it is getting clearer that the number of D4Z4 repeats at 4q35 or the *DUX4* misexpression do not *per se* fully characterize FSHD, and increasing efforts should be implemented to elucidate additional molecular or clinical features which could help patient stratification and promote the understanding of disease pathogenesis.

Here we examine the publications regarding FSHD over the last 18 months. The analysis was accomplished using search engines including Pubmed/Medline, Web of Science and Scopus, and generic keywords such as FSHD, 4q35 locus, FSHD treatment, FSHD models or more specific terms like DUX4, 4q35 methylation, epigenetics. 

**Box 1:**
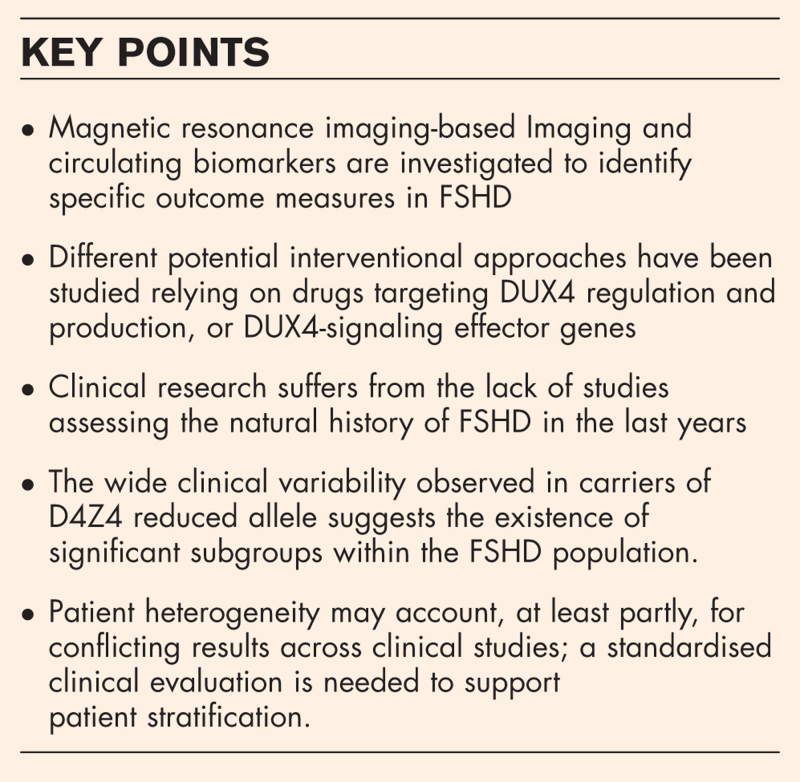
no caption available

## RESULTS

### Overview of FSHD literature reports

**FIGURE 1:**
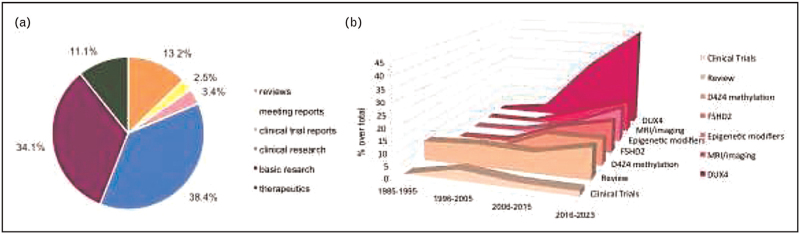
Main topics and trajectories of FSHD literature reports in the last 18 months. A. Pie chart representing the distribution of the selected papers as a percentage over the total (*n *= 121) based on the keywords reported in figure legend; B FSHD research trajectories over time. Data are plotted as a percentage of the total at different time points. Analysis was performed by considering 10-year timeframes between the first publication and today. Keywords are reported in the figure legend. FSHD, facioscapulohumeral muscular dystrophy.

Using the term FSHD, we selected 121 literature reports from July 2021 to January 2023. We subdivided them based on their type: reviews, meeting reports or research articles, and on the examined topics: clinical and epidemiological reports, clinical trial results, basic molecular research and therapeutics development. Figure 1A summarizes the number and the distribution of the selected papers: the large majority of publications regard basic research and clinical studies, 35.04% and 38.46%, respectively; reviews constitute 13.2% of all reports. Research on new potential therapeutic approaches occupies 3.42% and 10.26% of the whole.

In this time window, clinical research was mainly oriented on defining outcome measures to trace disease progression, such as improving muscle imaging by magnetic resonance imaging (MRI) or developing specific outcome measures. Efforts also aimed at exploring imaging and molecular biomarkers for their diagnostic and prognostic potential. Basic molecular research in the field was almost completely centered on DUX4 function and the evaluation of new genomic sequencing methods that could support the diagnostic process, including CpG methylation analyses. Figure 1B shows the literature trajectories over the years. From 1985 FSHD basic research has moved from the genetic and linkage analysis of 4q35 locus to the development of the DUX4-centered model, while the clinical approaches left away epidemiology and classical genotype/phenotype correlation in favor of patient stratification. based on MRI and proteomics. The percentage of promising clinical trials over the years remained unchanged.

### Clinical studies: are we looking in the right and same directions?

Over the last decade, the FSHD clinical field has focused on identifying disease biomarkers. This is because improved knowledge of the molecular basis of FSHD and results achieved in preclinical studies have fostered the development of new therapeutic strategies. Table 1 summarizes the main reports focused on prospective and retrospective clinical studies, with particular attention to the type of enrollment and the methodology applied for the clinical data collection, which might have a crucial role in the analysis. In the time window of this review, only a few studies investigated FSHD natural history and clinical variability, whereas the application of patient-reported outcome measures has expanded [18–20]. Two studies, one perspective regarding the US FSHD population and the other retrospective on Chinese cases, analyzed several parameters to define predictors of disease progression. Both studies established that disease duration is the most important predictor. However, the US study found that 27.3% of participants became wheelchair dependent [18], whereas a significantly lower percentage (8.9%) was reported in the Chinese cohort [19]. Both studies reported a faster rate of progression to wheelchair use in females. Besides the US and China studies on hundreds of participants, other studies on smaller cohorts confirmed the significant clinical variability among early-onset FSHD patients [20], including a wide spectrum of extramuscular manifestations, highlighting the need for careful monitoring of systemic symptoms by physicians [21]. A 5-year follow-up on respiratory function in FSHD patients showed that 44.6% presented a restrictive ventilatory pattern at baseline. However, only one-third developed a progressive respiratory dysfunction measured by forced vital capacity [22]. Two studies using self-reported measures, the Pittsburgh Sleep Quality Index and the Epworth Sleepiness Scale, respectively, found reduced sleep quality and excessive daytime sleepiness in FSHD patients and suggested that pain has a negative impact on sleep quality [23,24]. Two questionnaires, the coronavirus disease 2019 (COVID-19) Impact Survey and the Perceived Stress Scale, documented that the rates and the clinical outcome of COVID-19 infection in these patients were similar to the general population [25,26].

**Table 1:** Synopsis of the analysis and comparison of the major literature reports focused on clinical research in FSHD.

Article	Type	Participants	Follow up duration	Data collection duration	Outcome measures	Clinical evaluation during follow-up	Study enrollment
Katz *et al.* Brain. 2021 [18]	prospective	DRA *n *= 578	9 years average	2002–2019	Progression to wheelchair use	Self-reporting annual surveys	Registry based^a^
Wang *et al.*, Lancet Reg Health West Pac. 2021 [19]	prospective	DRA *n *= 1744; FSHD1 *n *= 971	10.5 years average	2001–2020	Clinical evaluation-muscle function	Yearly follow-up by telephone or video with neurologist	Clinician based^b^
Dijkstra *et al.*, Neurology. 2021 [20]	prospective	FSHD1 *n *= 20 children	2 years	2018–2020	Clinical evaluation-muscle function	Clinicians	Clinician based^c^
Teeselink S *et al.*, J Neurol. 2022 [22]	prospective	FSHD1 *n *= 88; FSHD2 *n *= 4	5 years	2014–2020	Respiratory function testing- clinicians	Clinicians, twice over five years.	Clinician based^d^
Eichinger *et al.* Muscle Nerve. 2021 [25]	prospective	FSHD *n *= 434; DMD *n *= 271; LGMD *n *= 69	preCovid/postCovid infection	May 2020	COVID-19 Impact Survey-self reported	NO	Registry based^e^
Lewis *et al.* J Neuromuscul Dis. 2022 [26]	prospective	FSHD *n *= 613; DMD *n *= 53; LGMD *n *= 99	precovid/postcovid infection	Febuary-March 2021	Improved COVID-19 Impact Survey -self reported	NO	Registry based^e^
Kelly *et al.*, Muscle Nerve. 2022 [21]	retrospective	FSHD1 *n *= 86; FSHD2 *n *= 1	/	/	Clinical evaluation-comorbidities self-reported	/	Clinician based^f^
Morse *et al.*, PLoS One 2022 [23]	retrospective	FSHD *n *= 12; BMD *n *= 15; LGMD *n *= 12	/	/	Sleep assessment- self reported	/	Clinician based^g^
Hoffmann *et al.*, Muscle Nerve 2022 [24]	retrospective	FSHD1 *n *= 352; FSHD2 *n *= 50; Unsure *n *= 249	/	/	Sleep assessment- self reported	/	Registry based^h^
Sanson *et al.*, Orphanet J Rare Dis. 2022 [27]	retrospective/ comparative	FSHD1 *n *= 281	/	/	Clinical evaluation-Clinician based and self-reported	/	Registry based^i^

a enrolled by The Registry, https://www.urmc.rochester.edu/neurology/national-registry.aspx.

b CCEF Categories; CSS and ACSS scores.

c MFM- MRC; NeuroQol fatigue; Kidscreen questionnaire.

d MFM- MRC;NeuroQol fatigue; Ricci score.

e enrolled via patient registries and patient advocacy groups.

f enrolled at Mayo Clinic (USA).

g enrolled at The Neuromuscular Centre-Winsford, UK.

h enrolled via Facioscapulohumeral Muscular Dystrophy Society-FSHDS.

i French National Registry of FSHD www.fshd.fr. BMD, Becker muscular dystrophy; DMD, Duchenne muscular dystrophy; DRA, D4Z4 reduced allele; FSHD, facioscapulohumeral muscular dystrophy; LGMD, limb girdle muscular dystrophy.

Remarkably, a French study assessing the concordance between a self-report questionnaire and a clinical evaluation form in FSHD found a good agreement only for demographic, diagnosis- and care-related items [27]. Instead, no concordance was found for muscle function-related features emphasizing that surveys alone should be used cautiously and complemented by objective medical data.

### Imaging and circulating biomarkers: diagnostic and prognostic potential

Great efforts have been directed towards identifying FSHD biomarkers in clinical research over the last 18 months, mainly imaging biomarkers, especially muscle MRI [28–32]. In a 1-year longitudinal study on 32 patients with FSHD, MRI-derived leg fat fraction was revealed to be a valid biomarker of disease progression [28]. A subsequent study documented that whole-body muscle MRI, especially scores for fat replacement and atrophy score, could be a potential biomarker of disease staging also in pediatric FSHD [30]. Recently in a longitudinal study on seventeen FSHD patients, a whole-body musculoskeletal MRI protocol was developed, and quantitative muscle measurements showed a strong correlation with functional outcome measures, including timed up-and-go (TUG) and FSHD-TUG [31]. A retrospective study demonstrated that muscle MRI could be a powerful diagnostic tool in differentiating FSHD from other inherited or acquired myopathies based on selective involvement of trapezius, sparing of subscapularis and iliopsoas, and asymmetric involvement of upper and lower limb muscles [32].

Tissue and circulating markers received little attention compared to image biomarkers, and no reliable biomarkers have been identified. Several groups found alterations in the circulating levels of several pro-inflammatory and regulatory cytokines (IL6) [33,34] or miRNAs that is, (miR-206) [35–38] and unsuccessfully investigated for the presence of disease-specific antibodies [39]. These molecules showed no disease specificity weakening their relevance for FSHD.

### Molecular basis of the disease: the DUX4 effect

The DUX4-driven model for FSHD assumes that DUX4 stochastic expression during muscle development triggers a toxic signaling cascade leading to muscle degeneration [40]. Banerji and Zammit [41^▪▪^] evaluated the role of DUX4 in FSHD pathogenesis by meta-comparing published data about the DUX4-signaling cascade. Their analysis indicated that the expression of DUX4 target gene is associated with muscle that exhibits inflammation. Instead, they connected the PAX7 signaling with persistent degeneration of FSHD skeletal muscle, even without overt inflammation. These, analyses suggest that DUX4, PAX7, and their interactions might be just two of the pieces of the FSHD puzzle.

The Zammit group further proved that the double homeobox 4 centromeric (*DUX4C*) is not just a pseudogene. Still, it is endogenously expressed at the protein level in normal and FSHD myotubes and in protein extracts of FSHD muscle biopsies [42,43]. The hDUX4C protein seems to be associated with muscle regeneration. This implies that DUX4 and DUX4c in regenerating FSHD muscle cells might have antagonistic roles and that caution should be exerted with therapeutic agents aiming for DUX4 suppression as they might also repress the highly similar DUX4c and interfere with its physiological role [44,45].

### New preclinical models for FSHD

Currently, the preclinical animal models for FSHD are prevalently represented by mice over-expressing high levels of DUX4. Because of the divergence of mDux and hDUX4, murine models have limitations that might explain their failure to recapitulate features of disease progression. Nip and colleagues [46] studied the porcine DUXC, showing that pDUXC and hDUX4 activate a highly similar early embryonic program in pig muscle cells. The same group also reported [47] the expression of two isoforms of DUXC mRNA in canine testis, showing that the canonical canine DUXC protein activates a cell signaling cascade similar to hDUX4 and mDux. Guo et colleagues [48] proposed using iPS -derived, induced iMyoblasts as an attractive model in several neuromuscular diseases. iMyoblast can be readily produced from FSHD patient iPSCs, and their selection protocol can recover a peculiar population of muscular cells with unique properties among other cellular models.

### Diagnosis and epigenetics in FSHD

Genetic diagnosis of FSHD is conventionally based on (i) determination of the D4Z4 repeat length and estimate of the repeat copy number of each array by Southern blotting, followed by (ii) confirmation of the presence of the 4qA allele, which constitutes a permissive haplotype. In cases without D4Z4 repeat contraction, *SMCHD1* gene and related epigenetic suppressor genes or D4Z4 CpG methylation are investigated. A nearly identical region complicates all this at the 10q26 subtelomere [49] and the numerous divergent D4Z4 arrays scattered throughout the genome favoring recombination events that may interfere with the capacity to interpret molecular results and generate a proper genotype-phenotype correlation. Furthermore, D4Z4 alleles with 4 to 8 repeats with 4qA can be found in approximately 2% of the general population [15].

In itself, the D4Z4 array structure impedes direct testing in preimplantation genetic diagnosis [50], and the highly recombinogenic nature of the 4q and 10q subtelomeres obstacles the use of alternative markers for PGD [51]. More recently, molecular combing [52–54] and optical mapping techniques (OGM) [55,56] have emerged to estimate the size of the array. In particular (OGM) has been applied to FSHD [55] for its ability to enumerate the repeats of the D4Z4 array on single long molecules of DNA. These methods have the potential to investigate structural variation associated with D4Z4 repeats throughout the entire genome, adding new capacity for interpreting uncommon molecular findings and phenotypes, even though they are technically and cost-effectively demanding, and none of them provides detailed information about the methylation status of the D4Z4 locus, a hallmark of FSHD.

Recently, Caputo and colleagues [57,58] developed a protocol for methylation analysis of specific CpG residues using iMachine Learning (ML) algorithms to classify FSHD cases. Hiramuki *et al.*, [59^▪^] applied long-read sequencing through a Nanopore CRISPR/Cas9-targeted resequencing to diagnose FSHD by simultaneous detection of D4Z4 repeat length and methylation status at the nucleotide level in FSHD patients. Erdmann *et al.*[60] developed a methylation-based diagnostic workflow comprising a haplotype and high-throughput methylation profile analyses (FSHD-MPA). FSHD-MPA determines the average global methylation level of the D4Z4 repeat array and the regional methylation of the most distal repeat unit by combining bisulfite conversion with next-generation sequencing and a bioinformatics pipeline and uses these as diagnostic parameters.

Although the D4Z4 CpG methylation has been extensively studied [61–66], the heterogeneity in methods and cohorts used in the different studies prevented a definite assessment of its contribution to the FSHD expression and severity. Indeed, Hiramuki *et al.*[59^▪^] recently observed that the hypomethylation in the contracted D4Z4 in FSHD1 is moderately correlated with patient phenotypes, while the Jones group [67] propose that the epigenetic status of the D4Z4 arrays can readily distinguish between healthy, FSHD1 and FSHD2 phenotypes. It can be anticipated that long-read sequencing applied to well clinically characterized cohorts will properly establish the clinical significance of reduced D4Z4 methylation.

### Clinical trials and new potential therapeutics

Over the decades, several therapeutic approaches have been attempted for FSHD. These included the administration of corticosteroids, beta2-adrenergic agonists, myostatin inhibitors, and oral supplementation with macronutrients and antioxidants to maintain muscle homeostasis and induce muscle hypertrophy, reviewed in [68]. Unfortunately, none of those nontargeted interventions achieved the primary outcome measures postulated in the study.

Therefore, more targeted approaches have been envisaged. Most of these attempts rely on drugs targeting DUX4 regulation and production or DUX4-signaling effector genes. They include: (i) epigenetic silencing of the D4Z4 repeats; (ii) blocking *DUX4* mRNA production; (iii) targeting downstream pathways triggered by *DUX4* expression as summarized by [68–72]. The main drug-based interventional studies applied to FSHD patients are summarized in Table 2. Notably, three out of six are still active, and two out of three are now recruiting FSHD patients.

**Table 2:** Description and comparison of drug based FSHD clinical trials started over years.

Drug/ ClinicalTrials.gov Identifier	Action	Responsible	Phase	Status	Start	End	Participants (n)
Losmapimod/ NCT05397470	P38 MAPK inhibitor	Fulcrum Therapeutics	PHASE 3	Recruiting	Jun 2022	Est. 2024	230
RO7204239/ GYM329/ NCT05548556	antimyostatin antibody	Hoffmann-La Roche	PHASE2	Recruiting	Feb 2023	Est. 2025	48
Losmapimod/ NCT04264442 NCT04004000	P38 MAPK inhibitor	Fulcrum Therapeutics	PHASE2	Active- Not Recruiting	2022 2019	Est. 2025 2024	76 14
Testosterone+ Somatropin/ NCT03123913	Hormone	University of Rochester	PHASE1	Completed	Feb 2019	Feb 2023	20
Losmapimod/ NCT04003974	P38 MAPK inhibitor	Fulcrum Therapeutics	PHASE2	Completed	Aug 2019	Jan 2021	80
ATYR1940 Resolaris/ NCT02603562 NCT02836418 NCT02531217 NCT02579239 NCT02239224	T-cells activation inhibitor	aTyr Pharma, Inc.	PHASE1/2 PHASE1/2 PHASE1/2 PHASE2 PHASE1/2	Completed	2016 2016 2015 2015 2014	2017 2017 2017 2017 2015	8 8 9 18 20
ACE-083/ NCT02927080 NCT03943290	Activins and myostatin inhibitor	Acceleron Pharma Inc.	PHASE2	Terminated Not achieving secondary endpoints	2020 2019	2022 2020	95 62

Est., estimated date; Part, Participants.

*LOSMAPIMOD*: developed by Fulcrum Therapeutics selectively inhibits the p38α/β mitogen-activated protein kinases (MAPKs), which are reported as modulators of DUX4 expression and mediators of inflammation [73,74]. A Phase 3 clinical trial using Losmapimod (ClinicalTrials.gov Identifier: NCT04264442) started in June 2022. Mellion *et al.*[73] summarized the results of Phase 1 trial supporting the advancing of Losmapimod into Phase 2. The drug was well tolerated, with no serious adverse effects. Data reported in June 2021 from the Phase IIb trial (ClinicalTrials.gov Identifier: NCT04003974), showed only a partial beneficial effect after 48 weeks of administration. The study's primary endpoint, the reduction of DUX4 mRNA and protein levels, was not met. It is to note that since p38 and MAPKs are known to regulate early stages of myogenesis, p38 inhibition may have adverse long-term effects in skeletal muscle, raising doubts on the likelihood of long-term benefit. These aspects should be carefully investigated in the next steps of the clinical trials.

*ACE-083*: Delivered by Acceleron Pharma [75–77], it mimics the ligand trap follistatin, and can bind both myostatin and activins. Although ACE-083 demonstrated a statistically significant increase in mean total muscle volume, which was the trial's (ClinicalTrials.gov Identifier: NCT02927080) functional endpoint, no significant improvements in functional tests were observed. As a result, Acceleron has recently decided to stop the clinical trial of ACE-083 in FSHD.

*DUX4 silencing strategies*: Several attempts at silencing DUX4 with small molecules, antisense therapeutics, genome editing techniques [78–85] or endogenous mi-RNAs are reported [86]. These attempts were performed *in vitro* and *in vivo* using cell lines or animal models overexpressing DUX4. This is because detecting DUX4 mRNA/protein in primary FSHD patient-derived muscle cells or muscle biopsies is difficult. In two different works [84,85], authors show that administering an octaguanidine dendrimer-conjugated phosphorodiamidate morpholino oligomer targeting DUX4–3’UTR, inhibits DUX4 expression and improves muscle functionality in DUX4 overexpressing mice [85]. In the same murine model, delivering a gapmer antisense oligonucleotide targeting DUX4-Orf also effectively knocked down DUX4 and alleviated muscle pathology, although with limited improvement in muscle mass and function [84].

Saad and colleagues [86] circumvented difficulties in delivering oligo-based RNAi systems by detecting a naturally DUX4 targeting human miRNA. They identified *miR-675* as a *DUX4* regulator, which directly targets *DUX4* mRNA, inhibiting its function. The delivery of *miR-675* by AAV-based gene therapy in scAAV6.CMV.DUX4-FL mice decreased DUX4-associated histopathology alterations.

## CONCLUSION

People living with FSHD experience progressive muscle weakness and atrophy that affect mobility, determine general disability, and impair social participation. We are still far from the optimal management of patients, and several responses are needed to address their actual demands. The review of publications in the past 18 months shows that despite the diverse approaches to tackling FSHD, diagnosis is often partial, and the individual prognosis remains uncertain. This lack of knowledge hinders designing therapeutics based on a specific target. At the same time, the demand for interventions is increasing as more people with FSHD age. It is a time for changes. We must elaborate new systems for data analysis that consider the clinical and genetic complexity of FSHD, support patient stratification and lay the basis for defining the disease's natural history associated with the distinct phenotypes.

**FIGURE 2:**
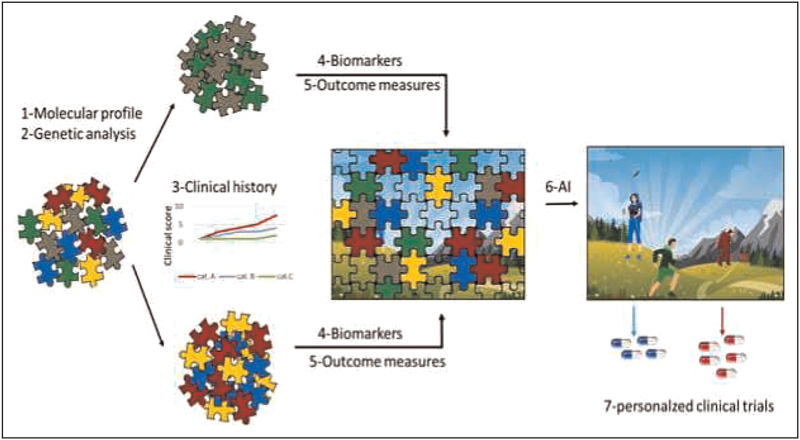
Solving the FSHD puzzle requires a multistep and multimodal approach. The wide phenotypic spectra observed in carriers of D4Z4 reduced allele suggest the existence of clinical subgroups within the FSHD population. Patients’ stratification relies on the combination of genomic, anamnestic, and standardised clinical data for defining disease natural history associated with the distinct phenotypes. These steps are necessary to develop appropriate outcome measures and biomarkers and to proceed toward personalized trial readiness for FSHD patients. FSHD, facioscapulohumeral muscular dystrophy.

A multidimensional platform exploiting advanced computational tools for systematically investigating FSHD cases might serve this goal (Fig. 2).

## Acknowledgements

*We are indebted to all FSHD patients and their families.*

### Financial support and sponsorship

*This work was supported by FAR2021-Mission Oriented*

### Conflicts of interest

*There are no conflicts of interest.*

## REFERENCES AND RECOMMENDED READING

Papers of particular interest, published within the annual period of review, have been highlighted as:

▪ of special interest
▪▪ of outstanding interest

## References

[1] Deutekom JCT va., Wljmenga C, Tlenhoven EAE va., *et al*. FSHD associated DNA rearrangements are due to deletions of integral copies of a 3.2 kb tandemly repeated unit. Hum Mol Genet [Internet]. 1993 [cited 2019 Sep 26]; 2:2037–42. Available from: http://www.ncbi.nlm.nih.gov/pubmed/8111371. 10.1093/hmg/2.12.2037 8111371

[2] Tremblay DC, Moseley S, Chadwick BP. Variation in array size, monomer composition and expression of the macrosatellite DXZ4. PLoS One [Internet]. 2011 [cited 2020 Apr 16]; 6:18969. Available from: www.plosone.org. 10.1371/journal.pone.0018969 PMC3081327 21544201

[3] Lemmers RJLF Van Der Vliet PJ Klooster R. A unifying genetic model for facioscapulohumeral muscular dystrophy. Science (80-) 2010; 329:1650–1653. 10.1126/science.1189044 PMC4677822 20724583

[4] Lemmers RJLF, Tawil R, Petek LM, *et al.* Digenic inheritance of an SMCHD1 mutation and an FSHD-permissive D4Z4 allele causes facioscapulohumeral muscular dystrophy type 2. Nat Genet [Internet]. NIH Public Access; 2012 [cited 2019 Sep 17]; 44:1370–4. Available from: http://www.ncbi.nlm.nih.gov/pubmed/23143600. 10.1038/ng.2454 PMC3671095 23143600

[5] Van Den Boogaard ML, Lemmers RJLF, Balog J, *et al.* Mutations in DNMT3B Modify Epigenetic Repression of the D4Z4 Repeat and the Penetrance of Facioscapulohumeral Dystrophy. Am J Hum Genet [Internet]. 2016 [cited 2019 Sep 17]; 98:1020–9. Available from: http://www.ncbi.nlm.nih.gov/pubmed/27153398. 10.1016/j.ajhg.2016.03.013 PMC4863565 27153398

[6] Jia FF Drew AP Nicholson GA. Facioscapulohumeral muscular dystrophy type 2: an update on the clinical, genetic, and molecular findings. Neuromuscul Disord 2021; 31:1101–12. 34711481 10.1016/j.nmd.2021.09.010

[7] Hamanaka K Šikrová D Mitsuhashi S. Homozygous nonsense variant in LRIF1 associated with facioscapulohumeral muscular dystrophy. Neurology 2020; 94:E2441–E2447. 32467133 10.1212/WNL.0000000000009617 PMC7455367

[8] Ricci G, Scionti I, Sera F, *et al.* Large scale genotype-phenotype analyses indicate that novel prognostic tools are required for families with facioscapulohumeral muscular dystrophy. Brain [Internet]. 2013 [cited 2019 Sep 18]; 136:3408–17. Available from: https://academic.oup.com/brain/article-lookup/doi/10.1093/brain/awt226. 10.1093/brain/awt226 PMC3808686 24030947

[9] Wu ZY Wang ZQ Murong SX Wang N. FSHD in Chinese population: characteristics of translocation and genotype-phenotype correlation. Neurology 2004; 63:581–583. 15304602 10.1212/01.wnl.0000133210.93075.81

[10] Goto K, Nishino I, Hayashi YK. Very low penetrance in 85 Japanese families with facioscapulohumeral muscular dystrophy 1A. J Med Genet [Internet]. 2004 [cited 2019 Sep 18]; 41:12e – 12. Available from: http://www.ncbi.nlm.nih.gov/pubmed/14729852. 10.1136/jmg.2003.008755 PMC1757263 14729852

[11] Nikolic A Ricci G Sera F. Clinical expression of facioscapulohumeral muscular dystrophy in carriers of 1-3 D4Z4 reduced alleles: experience of the FSHD Italian National Registry. BMJ Open 2016; 6:1–10. 10.1136/bmjopen-2015-007798 PMC4716236 26733561

[12] Nakagawa M, Matsuzaki T, Higuchi I, *et al.*Facioscapulohumeral Muscular Dystrophy: Clinical Diversity and Genetic Abnormalities in Japanese Patients. Intern Med [Internet]. 1997 [cited 2019 Sep 18]; 36:333–9. Available from: http://www.ncbi.nlm.nih.gov/pubmed/9213170. 10.2169/internalmedicine.36.333 9213170

[13] Sakellariou P, Kekou K, Fryssira H, *et al.* Mutation spectrum and phenotypic manifestation in FSHD Greek patients. Neuromuscul Disord [Internet]. 2012 [cited 2019 Sep 18]; 22:339–49. Available from: http://www.ncbi.nlm.nih.gov/pubmed/22357364. 10.1016/j.nmd.2011.11.001 22357364

[14] Salort-Campana E, Nguyen K, Bernard R, *et al.* Low penetrance in facioscapulohumeral muscular dystrophy type 1 with large pathological D4Z4 alleles: A cross-sectional multicenter study. Orphanet J Rare Dis [Internet]. BioMed Central; 2015 [cited 2019 Sep 18]; 10:2. Available from: http://www.ncbi.nlm.nih.gov/pubmed/25603992. 10.1186/s13023-014-0218-1 PMC4320820 25603992

[15] Scionti I Greco F Ricci G. Large-scale population analysis challenges the current criteria for the molecular diagnosis of fascioscapulohumeral muscular dystrophy. Am J Hum Genet [Internet] 2012; 90:628–635. 22482803 10.1016/j.ajhg.2012.02.019 PMC3322229

[16] Ricci G, Mele F, Govi M, *et al.* Large genotype–phenotype study in carriers of D4Z4 borderline alleles provides guidance for facioscapulohumeral muscular dystrophy diagnosis. Sci Rep [Internet]. Nature Research; 2020 [cited 2021 Feb 22]; 10:1–12. Available from: 10.1038/s41598-020-78578-7. PMC7730397 33303865

[17] Ricci G, Zatz M, Tupler R. Facioscapulohumeral Muscular Dystrophy: More Complex than it Appears. Curr Mol Med [Internet]. 2014 [cited 2019 Sep 6]; 14:1052–68. Available from: http://www.eurekaselect.com/openurl/content.php?genre=article&issn=1566-5240&volume=14&issue=8&spage=1052. 10.2174/1566524014666141010155054 PMC4264243 25323867

[18] Katz NK Hogan J Delbango R. Predictors of functional outcomes in patients with facioscapulohumeral muscular dystrophy. Brain 2021; 144:3451–3460. 34542603 10.1093/brain/awab326 PMC8677548

[19] Wang Z, Qiu L, Lin M, *et al.* Prevalence and disease progression of genetically-confirmed facioscapulohumeral muscular dystrophy type 1 (FSHD1) in China between 2001 and 2020: a nationwide population-based study. Lancet Reg Heal West Pacific [Internet]. Elsevier Ltd; 2022 [cited 2023 Apr 2]; 18:100323. Available from: http://www.ncbi.nlm.nih.gov/pubmed/35024656. 10.1016/j.lanwpc.2021.100323 PMC8671729 35024656

[20] Dijkstra JN Goselink RJM van Alfen N. Natural history of facioscapulohumeral dystrophy in children a 2-year follow-up. Neurology 2021; 97:E2103–E2113. 34675094 10.1212/WNL.0000000000012882 PMC8610619

[21] Kelly CR Saw JL Thapa P. Systemic manifestations and symptom burden of facioscapulohumeral muscular dystrophy in a referral cohort. Muscle Nerve 2022; 65:415–421. 35020192 10.1002/mus.27493

[22] Teeselink S Vincenten SCC Voermans NC. Long- term follow-up of respiratory function in facioscapulohumeral muscular dystrophy. J Neurol 2022; 269:3682–3689. 35147730 10.1007/s00415-022-10990-7 PMC8831680

[23] Morse CI Onambele-Pearson G Edwards B. Objective and subjective measures of sleep in men with Muscular Dystrophy. PLoS One 2022; 17. 10.1371/journal.pone.0274970 PMC9499246 36137167

[24] Hoffmann HM Malo-Juvera V Statland JM. Self-reported reduced sleep quality and excessive daytime sleepiness in facioscapulohumeral muscular dystrophy. Muscle Nerve 2022; 66:487–494. 35893768 10.1002/mus.27688 PMC9489670

[25] Eichinger K Lewis L Dilek N. A patient-focused survey to assess the effects of the COVID-19 pandemic and social guidelines on people with muscular dystrophy. Muscle Nerve 2021; 64:321–327. 34105174 10.1002/mus.27349 PMC8242695

[26] Lewis L Eichinger K Dilek N. Understanding the perseverance of the muscular dystrophy community one-year into the COVID-19 pandemic. J Neuromuscul Dis 2022; 9:517–523. 35723112 10.3233/JND-220794

[27] Sanson B Stalens C Guien C. Convergence of patient- and physician-reported outcomes in the French National Registry of Facioscapulohumeral Dystrophy. Orphanet J Rare Dis 2022; 17. 35236385 10.1186/s13023-021-01793-6 PMC8890461

[28] Wang LH Shaw DWW Faino A. Longitudinal study of MRI and functional outcome measures in facioscapulohumeral muscular dystrophy. BMC Musculoskelet Disord 2021; 22. 33691664 10.1186/s12891-021-04134-7 PMC7948347

[29] Deligianni X Santini F Paoletti M. Dynamic magnetic resonance imaging of muscle contraction in facioscapulohumeral muscular dystrophy. Sci Rep 2022; 12. 35508609 10.1038/s41598-022-11147-2 PMC9068910

[30] Woodcock IR de Valle K Varma N. Correlation between whole body muscle MRI and functional measures in paediatric patients with facioscapulohumeral muscular dystrophy. Neuromuscul Disord 2023; 33:15–23. 36522253 10.1016/j.nmd.2022.11.006

[31] Mellion ML Widholm P Karlsson M. Quantitative muscle analysis in FSHD using whole-body fat-referenced MRI: composite scores for longitudinal and cross-sectional analysis. Neurology 2022; 99:E877–E889. 35750498 10.1212/WNL.0000000000200757

[32] Monforte M Bortolani S Torchia E. Diagnostic magnetic resonance imaging biomarkers for facioscapulohumeral muscular dystrophy identified by machine learning. J Neurol 2022; 269:2055–2063. 34486074 10.1007/s00415-021-10786-1

[33] Muñoz-Cánoves P Scheele C Pedersen BK Serrano AL. Interleukin-6 myokine signaling in skeletal muscle: a double-edged sword? FEBS J 2013; 280:4131–4148. 23663276 10.1111/febs.12338 PMC4163639

[34] Gros M Nunes AM Daoudlarian D. Identification of serum interleukin 6 levels as a disease severity biomarker in facioscapulohumeral muscular dystrophy. J Neuromuscul Dis 2022; 9:83–93. 34459413 10.3233/JND-210711 PMC8842759

[35] Morton SU Sefton CR Zhang H. Microrna-mrna profile of skeletal muscle differentiation and relevance to congenital myotonic dystrophy. Int J Mol Sci 2021; 22:1–15. 10.3390/ijms22052692 PMC7962092 33799993

[36] García-Giménez JL García-Trevijano ER Avilés-Alía AI. Identification of circulating miRNAs differentially expressed in patients with Limb-girdle, Duchenne or facioscapulohumeral muscular dystrophies. Orphanet J Rare Dis 2022; 17. 36575500 10.1186/s13023-022-02603-3 PMC9793535

[37] Israeli D, Poupiot J, Amor F, *et al.* Circulating miRNAs are generic and versatile therapeutic monitoring biomarkers in muscular dystrophies. Sci Reports 2016 61 [Internet]. Nature Publishing Group; 2016 [cited 2023 Apr 7]; 6:1–11. Available from: https://www.nature.com/articles/srep28097. 10.1038/srep28097 PMC4914855 27323895

[38] Nunes AM Ramirez M Jones TI Jones PL. Identification of candidate miRNA biomarkers for facioscapulohumeral muscular dystrophy using DUX4-based mouse models. DMM Dis Model Mech 2021; 14. 10.1242/dmm.049016 PMC8405850 34338285

[39] Greco A Straasheijm KR Mul K. Profiling serum antibodies against muscle antigens in facioscapulohumeral muscular dystrophy finds no disease-specific autoantibodies. J Neuromuscul Dis 2021; 8:801–814. 34024774 10.3233/JND-210653 PMC9789485

[40] Mocciaro E, Runfola V, Ghezzi P, *et al*. DUX4 Role in Normal Physiology and in FSHD Muscular Dystrophy. Cells [Internet]. MDPI; 2021 [cited 2023 Apr 2];10. Available from: http://www.ncbi.nlm.nih.gov/pubmed/34943834. 10.3390/cells10123322 PMC8699294 34943834

[41▪▪] Banerji CRS Zammit PS. Pathomechanisms and biomarkers in facioscapulohumeral muscular dystrophy: roles of DUX4 and PAX7. EMBO Mol Med 2021; 13. 10.15252/emmm.202013695 PMC8350899 34151531

[42] Ganassi M, Figeac N, Reynaud M, *et al*. Antagonism Between DUX4 and DUX4c Highlights a Pathomechanism Operating Through β-Catenin in Facioscapulohumeral Muscular Dystrophy. Front cell Dev Biol [Internet]. Frontiers Media S.A.; 2022 [cited 2023 Apr 2]; 10:802573. Available from: http://www.ncbi.nlm.nih.gov/pubmed/36158201. 10.3389/fcell.2022.802573 PMC9490378 36158201

[43] Ansseau E, Eidahl JO, Lancelot C, *et al.* Transcription Factors DUX4 and DUX4c Associate with Cytoplasmic Proteins during Muscle Differentiation. PLoS One [Internet]. 2016 [cited 2023 Apr 14]; 11:146893. Available from: http://www.nih.gov/];. 10.1371/journal.pone.0146893 PMC4729438 26816005

[44] Claus C Slavin M Ansseau E. The double homeodomain protein DUX4c is associated with regenerating muscle fibers and RNA-binding proteins. Skelet Muscle 2023; 13. 36882853 10.1186/s13395-022-00310-y PMC9990282

[45] Bosnakovski D, Toso EA, Ener ET, *et al.* Antagonism among DUX family members evolved from an ancestral toxic single homeodomain protein. bioRxiv Prepr Serv Biol [Internet]. 2023 [cited 2023 Apr 2]; Available from: http://www.ncbi.nlm.nih.gov/pubmed/36711898. 10.1016/j.isci.2023.107823 PMC10514451 37744032

[46] Nip Y, Bennett SR, Smith AA, *et al.* Human DUX4 and porcine DUXC activate similar early embryonic programs in pig muscle cells: implications for preclinical models of FSHD. Hum Mol Genet [Internet]. Oxford University Press (OUP); 2023 [cited 2023 Apr 2]; Available from: http://www.ncbi.nlm.nih.gov/pubmed/36728804. 10.1093/hmg/ddad021 PMC10196675 36728804

[47] Wong CJ Whiddon JL Langford AT. Canine DUXC: Implications for DUX4 retrotransposition and preclinical models of FSHD. Hum Mol Genet 2022; 31:1694–1704. 34888646 10.1093/hmg/ddab352 PMC9122657

[48] Guo D Daman K Chen JJ. iMyoblasts for ex vivo and in vivo investigations of human myogenesis and disease modeling. Elife 2022; 11. 10.7554/eLife.70341 PMC8789283 35076017

[49] Van Overveld PGM, Lemmers RJFL, Deidda G, *et al.* Interchromosomal repeat array interactions between chromosomes 4 and 10: A model for subtelomeric plasticity. Hum Mol Genet [Internet]. 2000 [cited 2019 Sep 18];9:2879–84. Available from: http://www.ncbi.nlm.nih.gov/pubmed/11092764. 10.1093/hmg/9.19.2879 11092764

[50] Di Feo MF Bettio C Salsi V. Counseling and prenatal diagnosis in facioscapulohumeral muscular dystrophy: a retrospective study on a 13-year multidisciplinary approach. Heal Sci Rep 2022; 5:1–10. 10.1002/hsr2.614 PMC9059202 35509380

[51] Pini S Napoli FM Tagliafico E. De novo variants and recombination at 4q35: Hints for preimplantation genetic testing in facioscapulohumeral muscular dystrophy. Clin Genet 2023; 103:242–246. 36250762 10.1111/cge.14250 PMC10092082

[52] Nguyen K, Walrafen P, Bernard R, *et al.* Molecular combing reveals allelic combinations in facioscapulohumeral dystrophy. Ann Neurol [Internet]. 2011 [cited 2020 Jan 9];70:627–33. Available from: http://www.ncbi.nlm.nih.gov/pubmed/22028222. 10.1002/ana.22513 22028222

[53] Nguyen K, Puppo F, Roche S, *et al.* Molecular combing reveals complex 4q35 rearrangements in Facioscapulohumeral dystrophy. Hum Mutat [Internet]. 2017 [cited 2020 Jan 9];38:1432–41. Available from: http://www.ncbi.nlm.nih.gov/pubmed/28744936. 10.1002/humu.23304 28744936

[54] Nguyen K Broucqsault N Chaix C. Deciphering the complexity of the 4q and 10q subtelomeres by molecular combing in healthy individuals and patients with facioscapulohumeral dystrophy. J Med Genet 2019; 56:590–601. 31010831 10.1136/jmedgenet-2018-105949

[55] Koppikar P Shenoy S Guruju N Hegde M. Testing for facioscapulohumeral muscular dystrophy with optical genome mapping. Curr Protoc 2023; 3. 10.1002/cpz1.629 36648278

[56] Stence AA Thomason JG Pruessner JA. Validation of optical genome mapping for the molecular diagnosis of facioscapulohumeral muscular dystrophy. J Mol Diagnostics 2021; 23:1506–1514. 10.1016/j.jmoldx.2021.07.021 PMC8647435 34384893

[57] Caputo V Megalizzi D Fabrizio C. Update on the molecular aspects and methods underlying the complex architecture of FSHD. Cells 2022; 11. 36078093 10.3390/cells11172687 PMC9454908

[58] Caputo V Megalizzi D Fabrizio C. D4Z4 methylation levels combined with a machine learning pipeline highlight single CpG sites as discriminating biomarkers for FSHD patients. Cells 2022; 11. 36552879 10.3390/cells11244114 PMC9777431

[59▪] Hiramuki Y Kure Y Saito Y. Simultaneous measurement of the size and methylation of chromosome 4qA-D4Z4 repeats in facioscapulohumeral muscular dystrophy by long-read sequencing. J Transl Med 2022; 20. 36348371 10.1186/s12967-022-03743-7 PMC9644496

[60] Erdmann H, Scharf F, Gehling S, *et al.* Methylation of the 4q35 D4Z4 repeat defines disease status in facioscapulohumeral muscular dystrophy. Brain [Internet]. Oxford University Press (OUP); 2022 [cited 2023 Apr 2]; Available from: http://www.ncbi.nlm.nih.gov/pubmed/36100962. 10.1093/brain/awac336 36100962

[61] Salsi V, Magdinier F, Tupler R. Does DNA methylation matter in FSHD? Genes (Basel) [Internet]. [2020] [cited 2020 Mar 4];11:258. Available from: https://www.mdpi.com/2073–4425/11/3/258. 10.3390/genes11030258 PMC7140823 32121044

[62] Nikolic A, Jones TI, Govi M, *et al.* Interpretation of the epigenetic signature of facioscapulohumeral muscular dystrophy in light of genotype-phenotype studies. Int J Mol Sci [Internet]. Int J Mol Sci; 2020 [cited 2020 Apr 16];21:2635. Available from: https://www.mdpi.com/1422-0067/21/7/2635. 10.3390/ijms21072635 PMC7178248 32290091

[63] de Greef JC, Lemmers RJLF, van Engelen BGM, *et al.*Common epigenetic changes of D4Z4 in contraction-dependent and contraction-independent FSHD. Hum Mutat [Internet]. John Wiley & Sons, Ltd; 2009 [cited 2019 Oct 7];30:1449–59. Available from: http://doi.wiley.com/10.1002/humu.21091. 10.1002/humu.21091 19728363

[64] Calandra P, Cascino I, Lemmers RJLF, *et al.* Allele-specific DNA hypomethylation characterises FSHD1 and FSHD2. J Med Genet [Internet]. BMJ Publishing Group; 2016 [cited 2019 Oct 7];53:348–55. Available from: http://www.ncbi.nlm.nih.gov/pubmed/26831754. 10.1136/jmedgenet-2015-103436 26831754

[65] Lemmers RJLF, Goeman JJ, Van der Vliet PJ, *et al.* Inter-individual differences in CpG methylation at D4Z4 correlate with clinical variability in FSHD1 and FSHD2. Hum Mol Genet [Internet]. Oxford University Press; 2015 [cited 2019 Oct 7];24:659–69. Available from: http://www.ncbi.nlm.nih.gov/pubmed/25256356. 10.1093/hmg/ddu486 PMC4291246 25256356

[66] Huichalaf C Micheloni S Ferri G. DNA methylation analysis of the macrosatellite repeat associated with FSHD muscular dystrophy at single nucleotide level. PLoS One 2014; 9. 10.1371/journal.pone.0115278 PMC4278900 25545674

[67] Gould T, Jones TI, Jones PL. Precise Epigenetic Analysis Using Targeted Bisulfite Genomic Sequencing Distinguishes FSHD1, FSHD2, and Healthy Subjects. Diagnostics (Basel, Switzerland) [Internet]. MDPI; 2021 [cited 2023 Apr 2];11. Available from: http://www.ncbi.nlm.nih.gov/pubmed/34441403. 10.3390/diagnostics11081469 PMC8393475 34441403

[68] Wang LH Tawil R. Current Therapeutic Approaches in FSHD. J Neuromuscul Dis 2021; 8:441–451. 33579868 10.3233/JND-200554 PMC8203219

[69] Himeda CL, Jones PL. FSHD Therapeutic Strategies: What Will It Take to Get to Clinic? J Pers Med [Internet]. MDPI; 2022 [cited 2023 Apr 2];12. Available from: http://www.ncbi.nlm.nih.gov/pubmed/35743650. 10.3390/jpm12060865 PMC9225474 35743650

[70] Tihaya MS Mul K Balog J. Facioscapulohumeral muscular dystrophy: the road to targeted therapies. Nat Rev 2023; 19:91–108. 10.1038/s41582-022-00762-2 PMC11578282 36627512

[71] Cohen J DeSimone A Lek M Lek A. Therapeutic approaches in facioscapulohumeral muscular dystrophy. Trends Mol Med 2021; 27:123–137. 33092966 10.1016/j.molmed.2020.09.008 PMC8048701

[72] Schwarzkopf M Coletti D Sassoon D Marazzi G. Muscle cachexia is regulated by a p53- PW1/Peg3-dependent pathway. Genes Dev 2006; 20:3440–3452. 17182869 10.1101/gad.412606 PMC1698450

[73] Mellion ML Ronco L Berends CL. Phase 1 clinical trial of losmapimod in facioscapulohumeral dystrophy: safety, tolerability, pharmacokinetics, and target engagement. Br J Clin Pharmacol 2021; 87:4658–4669. 33931884 10.1111/bcp.14884

[74] Brennan CM Hill AS St. Andre M. DUX4 expression activates JNK and p38 MAP kinases in myoblasts. DMM Dis Model Mech 2022; 15. 10.1242/dmm.049516 PMC10655719 36196640

[75] Statland JM, Campbell C, Desai U, *et al.* Randomized phase 2 study of ACE-083, a muscle-promoting agent, in facioscapulohumeral muscular dystrophy. 2022 [cited 2023 Apr 2]; 66:50–62. Available from: https://pubmed.ncbi.nlm.nih.gov/35428982/. 10.1002/mus.27558 PMC9321022 35428982

[76] Pearsall RS Davies MV Cannell M. Follistatin-based ligand trap ACE-083 induces localized hypertrophy of skeletal muscle with functional improvement in models of neuromuscular disease. Sci Rep 2019; 9. 31388039 10.1038/s41598-019-47818-w PMC6684588

[77] Glasser CE, Gartner MR, Wilson D, *et al*. Locally acting ACE-083 increases muscle volume in healthy volunteers. Muscle Nerve [Internet]. Muscle Nerve; 2018 [cited 2023 Apr 14];57:921–6. Available from: https://pubmed.ncbi.nlm.nih.gov/29486514/. 10.1002/mus.26113 PMC5969095 29486514

[78] Rashnonejad A, Amini-Chermahini G, Taylor NK, *et al.* Designed U7 snRNAs inhibit DUX4 expression and improve FSHD-associated outcomes in DUX4 overexpressing cells and FSHD patient myotubes. Mol Ther Nucleic Acids [Internet]. Cell Press; 2021 [cited 2023 Apr 2];23:476–86. Available from: http://www.ncbi.nlm.nih.gov/pubmed/33510937. 10.1016/j.omtn.2020.12.004 PMC7807095 33510937

[79] Lim KRQ, Yokota T. Genetic Approaches for the Treatment of Facioscapulohumeral Muscular Dystrophy. Front Pharmacol [Internet]. Frontiers Media S.A.; 2021 [cited 2023 Apr 2];12:642858. Available from: http://www.ncbi.nlm.nih.gov/pubmed/33776777. 10.3389/fphar.2021.642858 PMC7996372 33776777

[80] Lim KRQ Yokota T. Knocking Down DUX4 in Immortalized Facioscapulohumeral Muscular Dystrophy Patient-Derived Muscle Cells. Methods Mol Biol 2023; 2587:197–208. 36401032 10.1007/978-1-0716-2772-3_12

[81] Bouwman LF, den Hamer B, van den Heuvel A, *et al.*Systemic delivery of a DUX4-targeting antisense oligonucleotide to treat facioscapulohumeral muscular dystrophy. Mol Ther Nucleic Acids [Internet]. Cell Press; 2021 [cited 2023 Apr 2]; 26:813–27.Available from: http://www.ncbi.nlm.nih.gov/pubmed/34729250. 10.1016/j.omtn.2021.09.010 PMC8526479 34729250

[82] Himeda CL, Jones TI, Jones PL. Targeted epigenetic repression by CRISPR/dSaCas9 suppresses pathogenic DUX4-fl expression in FSHD. Mol Ther Methods Clin Dev [Internet]. Cell Press; 2021 [cited 2023 Apr 2]; 20:298–311. Available from: http://www.ncbi.nlm.nih.gov/pubmed/33511244. 10.1016/j.omtm.2020.12.001 PMC7806950 33511244

[83] Das S Chadwick BP. CRISPR mediated targeting of DUX4 distal regulatory element represses DUX4 target genes dysregulated in Facioscapulohumeral muscular dystrophy. Sci Rep 2021; 11. 34131248 10.1038/s41598-021-92096-0 PMC8206090

[84] Lu-Nguyen N Malerba A Herath S. Systemic antisense therapeutics inhibiting DUX4 expression ameliorates FSHD-like pathology in an FSHD mouse model. Hum Mol Genet 2021; 30:1398–1412. 33987655 10.1093/hmg/ddab136 PMC8283208

[85] Lim KRQ Bittel A Maruyama R. DUX4 transcript knockdown with antisense 2′-O-methoxyethyl gapmers for the treatment of facioscapulohumeral muscular dystrophy. Mol Ther 2021; 29:848–858. 33068777 10.1016/j.ymthe.2020.10.010 PMC7854280

[86] Saad NY Al-Kharsan M Garwick-Coppens SE. Human miRNA miR-675 inhibits DUX4 expression and may be exploited as a potential treatment for Facioscapulohumeral muscular dystrophy. Nat Commun 2021; 12. 34880230 10.1038/s41467-021-27430-1 PMC8654987

